# Bacterial community composition responds to changes in copepod abundance and alters ecosystem function in an Arctic mesocosm study

**DOI:** 10.1038/s41396-018-0217-7

**Published:** 2018-07-10

**Authors:** Tatiana M. Tsagaraki, Bernadette Pree, Øystein Leiknes, Aud Larsen, Gunnar Bratbak, Lise Øvreås, Jorun K. Egge, Roman Spanek, Maria L. Paulsen, Yngvar Olsen, Olav Vadstein, T. F. Thingstad

**Affiliations:** 1 Department of Biological Sciences, University of Bergen, Bergen, Norway; 2 Department of Biology, NTNU Norwegian University of Science and Technology, Trondheim, Norway; 3 Uni Research Environment, Uni Research AS, Bergen, Norway; 4 The Institute of Mechatronics and Computer Engineering, Technical University of Liberec, Liberec, Czech Republic; 5 Department of Biotechnology and Food Science, NTNU Norwegian University of Science and Technology, Trondheim, Norway; 6 Present Address: The University Centre in Svalbard, Longyearbyen, Norway

**Keywords:** Microbial ecology, Microbial ecology

## Abstract

Combining a minimum food web model with Arctic microbial community dynamics, we have suggested that top-down control by copepods can affect the food web down to bacterial consumption of organic carbon. Pursuing this hypothesis further, we used the minimum model to design and analyse a mesocosm experiment, studying the effect of high (+Z) and low (-Z) copepod density on resource allocation, along an organic-C addition gradient. In the Arctic, both effects are plausible due to changes in advection patterns (affecting copepods) and meltwater inputs (affecting carbon). The model predicts a trophic cascade from copepods via ciliates to flagellates, which was confirmed experimentally. Auto- and heterotrophic flagellates affect bacterial growth rate and abundance via competition for mineral nutrients and predation, respectively. In +Z, the model predicts low bacterial abundance and activity, and little response to glucose; as opposed to clear glucose consumption effects in –Z. We observed a more resilient bacterial response to high copepods and demonstrate this was due to changes in bacterial community equitability. Species able to use glucose to improve their competitive and/or defensive properties, became predominant. The observed shift from a SAR11-to a *Psychromonodaceae* – dominated community suggests the latter was pivotal in this modification of ecosystem function. We argue that this group used glucose to improve its defensive or its competitive abilities (or both). Adding such flexibility in bacterial traits to the model, we show how it creates the observed resilience to top-down manipulations observed in our experiment.

## Introduction

The microbial food web in the photic zone plays a central role in the oceans’ ecosystem functions, such as the cycling of mineral nutrients [1] and oceanic carbon sequestration [2]. It is predicted that Arctic ecosystems will be among the most affected by climate change [3] as freshening, warming and acidification are occurring faster there than in other regions [4–6]. Considering that input of fluvial [7] and glacial [8, 9] dissolved organic matter (DOM) and seasonal migration of copepods [10] are central characteristics of the Arctic Ocean, it is highly relevant to understand how (marine) organisms and ecosystem function are coupled in the context of a rapidly changing Arctic [11].

Aristotle’s concept of synergy is summarised as “The whole is more than the sum of its parts*”* and this is particularly true for the oceans role in global carbon cycling. Build-up of carbon through the classical food chain contributes to the production of sinking particles that fuel the Biological Pump (BP) [12, 13]. Additionally, a large part of dissolved organic carbon (DOC) consumed by microbes is converted into recalcitrant forms via the microbial carbon pump (MCP) [14, 15]. Carbon is sequestered through the BP and the MCP simultaneously and it’s rate is hypothesised to vary based on the trophic state of the system [14, 16].

The connection of ecosystem function to diversity, life strategy and trophic interactions is a major challenge in marine microbiology [17] and in order to gain a predictive understanding of community dynamics and function, the integration of theory with models and experimental data is required [18]. Several studies look at these aspects for selected groups, e.g. bacterial diversity and carbon cycling [19], predator life strategy and carbon sequestration [20], competition within phytoplankton groups at different CO_2_ conditions [21]. Few studies address these questions at the food web level (e.g. [22, 23]). Both these studies suggest that the community dynamics and structure play a pivotal role in the form and quantity of food web mediated export.

In an attempt to understand how trophic interactions affect the structure and function of the microbial food web we have used a ‘minimum’ mathematical food web model [24] to interpret the results of mesocosm experiments. The minimum model has so far reproduced observed system responses of two bottom-up manipulation experiments in Ny Ålesund, Svalbard. However, model and experimental results have suggested that copepod abundance is a major influence in the structure and function of Arctic food webs [25]. Further, the initial copepod standing stock has also been suggested to affect bacterial and virus communities, where high copepods were connected to mineral nutrient limited heterotrophic prokaryotes while low copepods associated with carbon limited heterotrophic prokaryotes [26]. The latter study provides an example of how rapid dynamics of the trophic interactions within the microbial food web created strong coupling through ca. 5 orders of magnitude in organism size (copepods –viruses), affecting nutrients and carbon cycling. Even though our minimum model has pointed to the regulating effect of top predators, the predator community has not been manipulated experimentally before.

**Fig. 1:**
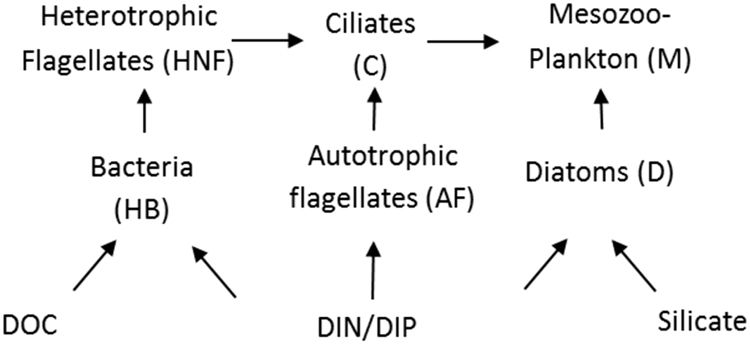
Minimum food web model redrawn from Thingstad et al. [24]. Dissolved organic carbon (DOC) and mineral nutrients, such as silicate, inorganic nitrogen and phosphate (DIN, DIP) enter the food web via osmotrophic communities, which are preyed upon by phagotrophic communities

**Fig. 2:**
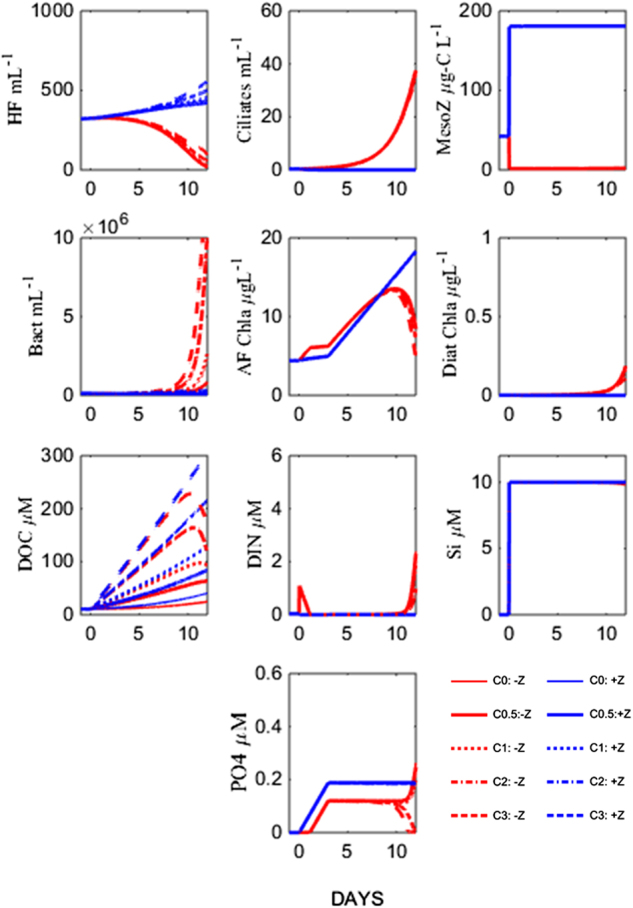
Hypothesised responses of abundances and biomasses of microbial communities represented in the minimum food web model [24] to copepod and carbon manipulations. The blue and red lines represent Z+ and Z- treatments, respectively. The glucose gradients are indicated by line thickness increasing with increasing glucose additions

To test our hypothesis of strong top predator control on organic carbon and mineral nutrient cycling in the marine microbial food web, we designed a new mesocosm experiment in an Arctic location (Ny Ålesund, Svalbard) where we contrasted high and low copepod abundance along a gradient of carbon additions. The “minimum” food web model (Fig. 1), was used to model responses for each mesocosm (Fig. 2). This model output represents our hypotheses for the outcome of the experiment and can be summarised as:

- High copepod abundance (+Z) triggers a trophic cascade where low ciliate abundance, releases the grazing pressure on auto- and heterotrophic flagellates. The two flagellate populations constrain bacterial abundance through increased mineral nutrient competition and predation, respectively, resulting in reduced glucose consumption which means no differences in bacterial abundance are expected along the carbon gradient.
- Removal of mesozooplankton grazers (−Z), leads to high ciliate abundance, creating conditions with low mineral nutrient competition and low grazing pressure for bacteria. The expected results are high bacterial abundance and glucose consumption, and consequently that bacterial abundance will scale to the amount of glucose addition. Combined with silicate addition, removal of the copepod grazers creates a niche for diatoms, which in Fig. 2 appear in the –Z treatments.

## Materials and Methods

### Mesocosm setup and manipulations

The mesocosm experiment was conducted in Kings Bay on the east coast of Svalbard from 26th June 8th July 2015. We used ten 1250 L high-density translucent polyethylene tanks (Ecobulk MX; Schütz, Selters, Germany) as experimental units. The filling procedure and copepod manipulations are described in detail in the SI.

At start of the experiment (after time zero sampling) we added 5 adult copepods per litre to one set of five tanks (+Z tanks) while the remaining five tanks were kept at low initial copepod abundance (−Z tanks). Nutrients, i.e. ammonia, phosphate and glucose in aqueous stocks was added daily from day one, resulting in two carbon to mineral nutrient ratio gradients (one for +Z and one for –Z) with *C* = 0, 0.5, 1, 2, and 3 × Redfield (1 × Redfield = 7.4 µM C, 1.12 µM N and 0.07 µM P). Si was added in surplus (11.4 µM) on day 1 and after that 1.2 µM Si was added every second day to maintain it in surplus. The tanks were accordingly denoted –Z: 0C–3C and +Z: 0C–3C.

### Analysis of dissolved constituents

Dissolved inorganic phosphate, silicate, and ammonium were measured immediately after sampling according to the methods described in Koroleff [27], Valderrama [28], and Holmes et al. [29], respectively.

Samples for total organic carbon (TOC) were collected in 30 ml acid washed high-density polyethylene bottles and stored frozen (−20 °C) until analysis. TOC concentrations were determined by high temperature combustion (720 °C) using a Shimadzu TOC-V CPH-TN carbon and nitrogen analyser (Shimadzu Corporation) according to Cauwet [30]. The instrument was calibrated using a standard series of acetoanilide and quality controlled using carbon reference material provided by the Hasell consensus reference material programme (Hansell Lab, U. Miami, USA).

Particulate organic carbon (POC) samples were filtered in triplicates onto pre-combusted (450 °C, overnight), 25 mm, GF/F filters (Whatman). Filtration volumes varied between 100 and 400 ml, filters were kept frozen (−20 °C) until analysis. The filters were fumed with saturated HCl and analysed according to Pella and Colombo [31] in a Flash 2000 elemental analyser (Thermo Fisher Scientific, Waltham, MA, USA).

DOC was calculated as TOC minus POC from day 3 onwards.

### Quantification of abundances and biomasses

On day zero, subsamples for mesozooplankton were collected. Samples from the –Z tanks (day zero and 13), and from the +Z tanks (day 13) were collected by pumping 80–100 L of water from the tanks while they were mixed. This water was then filtered using a 20 µm plankton sieve. Representatives of the genus *Calanus* were identified to species based on morphology and prosome lengths of individual copepodite stages based on the size distribution by Kwasniewski et al. [32]. Biomass was calculated using length–dry weight conversions from Hay et al. [33] and Hay et al. [34] assuming a carbon content of 45 % [35].

Ciliate abundance was quantified using a black and white imaging FlowCAM® II (Fluid Imaging Technologies, Maine, USA). Fresh samples were run for 30 min using a 10 × objective, in Auto Image mode. Ciliates and dinoflagellates were sorted manually by visual inspection of the image database.

Heterotrophic nanoflagellates (HNF) and bacteria was determined using an Attune® Acoustic Focusing Flow Cytometer (Applied Biosystems by Life technologies) with a syringe-based fluidic system and a 20 mW 488 nm (blue) laser. Samples were fixed with glutaraldehyde (0.5% final conc.), flash frozen in liquid nitrogen and stored at −80 °C. Prior to analyses samples were thawed and stained with SYBR Green I (Molecular Probes, Eugene, Oregon, USA) for a minimum of 1 h. Heterotrophic bacteria were analysed using a flow rate of 25 µl min^−1^ and HNF at 500 µl min^−1^, following protocols modified from Marie et al. [36] and Zubkov et al. [37]. For bacteria, flow cytometer plots showed two distinct populations, corresponding to different (high and low) nucleic-acid staining properties. Briefly, these populations are discriminated based the red and green fluorescence after staining with SYBR green. The stain reflects the amount of DNA in each cell and these, referred to as HNA and LNA bacteria, are attributed different properties connected to activity and size [38–45]. From day 7–8 of the experiment, a subpopulation of HNA, with higher red and green fluorescence signal, appear in carbon-amended tanks (Fig. S2). Relative abundance of this subgroup abundance was calculated in all tanks throughout the experiment.

Chlorophyll *a* was filtered onto 47 mm Nuclepore filters and analysed fluorometrically according to Holm-Hansen and Riemann [46]. Total chl *a* (>0.2 µm) was filtered in triplicates every second day and fractionated chl *a* on filters of pore sizes 0.2, 0.8 and 10 μm on the days between total chl *a*. Chl *a* was extracted in methanol at 4 °C overnight before measuring on a Turner Designs 10-AU Fluorometer calibrated with pure chl *a* (Sigma Chemicals Inc.).

### Determination of biological rates

The production of biomass by heterotrophic bacteria was quantified by incorporation of ^3^H leucine into macromolecules (protein biosynthesis). Radioactive leucine was added to the samples at a final concentration of 40 nM, incubated for 1 h at ambient temperature (±1 °C), stopped by addition of trichloracetic acid, and processed by the centrifugation method [47]. Incorporation of ^3^H was quantified by liquid scintillation counting (PerkinElmer Life and Analytical Sciences, Waltham, MA, USA), and the production of biomass by heterotrophic bacteria was quantified on a carbon basis in accordance with Kirchman [48] assuming no isotope dilution.

### Molecular analysis of bacterial community composition

Samples for molecular analyses focused on 6 of the 10 treatments, 3 tanks with low copepod abundance (−Z: 0C, 1C, 3C) and 3 tanks with high copepod abundance (+Z: 0C, 1C, 3C). Samples were collected every second day onto 0.2 µm Sterivex filters (1 – 3l). The V4 region of the 16S rRNA gene was used for the sequence based amplification generation, with indexing and sequencing of amplicon libraries using the MiSeq Platform at the Norwegian Sequencing Centre (Oslo, Norway). See SI for more details on sample storage, extraction, amplification and sequencing.

### Bioinformatic analysis

The 16S rRNA sequences were processed using Mothur [49], including quality filtering and chimera check using the Pyro Noise implemented in Mothur [50] and chimera UCHIME [51]. The sequences were aligned against the SILVA database (version 123). Mitochondrial and chloroplast sequences were removed prior to OTU construction. Alignments were constructed using Needleman-Wunsch pairwise alignment method implemented as align.seqs command in Mothur [52]. All OTUs were constructed using a 97% sequence identity cut-off. Singletons were removed before clustering and all singletons were subsequently linked to taxonomy file in order to know what kind of sequences that were cut from the datasets. Size (number of sequences) of all samples was normalised to the size of the smallest sample [53].”

### Statistical analysis

Trophic cascades are defined as a negative correlation between the biomasses or abundances of adjacent trophic levels and a positive one between trophic levels two links apart ([54, 55], reviewed by [56]). We run a Pearson correlation (IBM SPSS) between abundance of ciliates, total chl *a* biomass (as a proxy for autotrophic flagellates*)*, and HNF and bacteria abundance on days 3, 5, 7, 9, 11. The trophic links assumed follow Fig. 1, excluding diatoms. We pooled all treatments and days together because the trophic cascade should work across both + and −Z in the same way.

### Model description

The mathematical formulation of the ‘minimum’ microbial food web model (Fig. 1) is described in detail in Thingstad et al. [24]. Briefly, the model consists of six plankton functional types (PFT) and uses phosphorus (P) as the basic unit. Specific growth rates are defined as hyperbolic curves defined by their maximum value (µ^max^) and slope at 0 resource concentration (*α*), i.e. representing maximum clearance rate for phagotrophs and maximum phosphate affinity for osmotrophs. A yield parameter is associated with each phagotroph, defining how much of the P of prey biomass, ends up in predator biomass, the surplus is assumed to be instantaneously remineralised to free orthophosphate. Bacterial and diatom growth can either be limited by P, or by biodegradable DOC and by free silicate, respectively. The original set of parameters [24], established for a system with higher tempereture (ca 10 °C), was used but all µ^max^ and *α* values are divided by 1.4 to include the temperature correction suggested by Larsen et al. [25].

The initial state was calculated by assuming the microbial part to be in steady state. This steady state is a function of the amount of mesozooplankton (Z_0_), the total-P available to the microbial part (P_T0_), and the model parameters. Calculation of the initial steady state is simplified by assuming all food uptakes to be proportional to food concentration. This approximation to steady state becomes better the more food limited the organisms are.

At time = 0, the mesozooplankton stocks in +Z were changed and nutrient additions initiated, mimicking the different mesocosm treatments. The simulated P concentrations were converted to units comparable with observations using fixed conversion factors. Further details of the model, parameters and conversion factors are given in SI.

## Results

### Abundances, biomasses

Correlation analysis of abundances of ciliates, HNF, bacteria and total chl *a* concentrations revealed presence of a trophic cascade from copepods to bacteria with significant negative correlations between ciliates and their prey (HNF and total chl *a*) and a negative one, although not significant, between HNF and their prey (bacteria) (Table 1). There was also a statistically significant positive correlation between ciliates and bacteria.

**Table 1:** Pearson correlation coefficient and significance levels between ciliates, HNF, Chla and bacteria for all tanks and experimental days

Pearson correlation				
	Ciliates	HNF	Bacteria	Chla
*Ciliates*				
Pearson correlation	1	−0.620^**^	0.582^**^	−0.477^**^
Sig. (2-tailed)		0	0	0
*N*	50	50	50	50
*HNF*				
Pearson correlation	−0.620^**^	1	−0.048	0.796^**^
Sig. (2-tailed)	0		0.741	0
*N*	50	50	50	50
*Bacteria*				
Pearson correlation	0.582^**^	−0.048	1	−0.001
Sig. (2-tailed)	0	0.741		0.993
*N*	50	50	50	50
*Chla*				
Pearson correlation	−0.477^**^	0.796^**^	−0.001	1
Sig. (2-tailed)	0	0	0.993	
*N*	50	50	50	50

**Correlation is significant at the 0.01 level (2-tailed).

Abundances of microbial communities, mesozooplankton biomass, concentrations of chl *a*, DOC and mineral nutrients over the course of the experiment are shown in Fig. 4. Where mesozooplankton > 200μm were removed (−Z), their biomass decreased from 22 µg C l^−1^ at the start of the experiment to 5 ± 2 µg C l^−1^ (mean ± SD) at the end. Ciliates increased rapidly from day 3 onwards, from initial abundances of less than 1 ciliate per ml to 118 ± 24 ciliates per ml on day 11. Heterotrophic nanoflagellate initial abundance was 620 ± 65 cells per ml and increased to 1280 ± 144 cells per ml on day 1. The abundance of HNF decreased to less than 50 cells per ml in 0 C tanks towards the end of the experiment. In tanks with C additions, HNF increased to around 500 cells per ml towards the end. Bacteria abundance was 0.8 ± 0.0 × 10^6^ cells per ml and remained stable until day 8. After that, bacteria increased to peak abundances of 4.9 ± 0.9 × 10^6^ cells per ml, with a maximum in tanks with 1 C addition (5.8 × 10^6^ cells per ml) and minimum in the 0 C tank (3.4 × 10^6^ cells per ml). Autotrophs were dominated by the fraction of cells < 10 µm throughout the experiment and were not affected by carbon additions. Total chl *a* concentration remained at levels similar to initial values (1.6 ± 0.0 µg chl *a* l^−1^) for 5 days and after a peak of 3.0 ± 0.3 µg chl *a* l^−1^ it decreased to initial concentrations.

In + Z tanks, the final biomass of mesozooplankton (300 ± 110 µg C l^−1^) was more than tenfold higher than initial biomass (22 µg C l^−1^). The added copepods consisted of *Calanus finmarchicus* and *C. glacialis* copepodites, stage 4 and 5. Analysis of the –Z tanks at the end of the experiment showed some small copepods, consisting mainly of copepodite nauplii and *Oithona* sp. Ciliates remained at abundances less than 1 cell per ml throughout the experiment which allowed for HNF to increase from 620 ± 78 cells per ml to approximately 3000 cells per ml on days 8–9. The abundance of HNF was slightly lower in 0 C tanks, with a maximum of 2690 cells per ml at the end of the experiment. Bacteria decreased to half of their initial abundances of 0.8 ± 0.0 × 10^6^ cells per ml until day 7. Thereafter it increased to a maximum of 2.5 ± 0.4 × 10^6^ cells per ml, with the lowest abundance in 0 C tanks (2.2 × 10^6^ cells per ml). The autotrophic community was dominated by cells < 10 µm and increased throughout the experiment. The bloom had highest maximum concentrations of 11.7 µg chl *a* l^−1^ in 0 C tanks. Total chl *a* levelled off at 7.6 ± 0.5 µg chl *a* l^−1^ in the other tanks at the end of the experiment. Chysophyseas (*Meringosphaera* sp) and small naked dinoflagellates, in addition unidentified flagellates dominated the phytoplankton community >10 µm (J. Egge, personal communication).

### Mineral nutrients, DOC

**Fig. 3:**
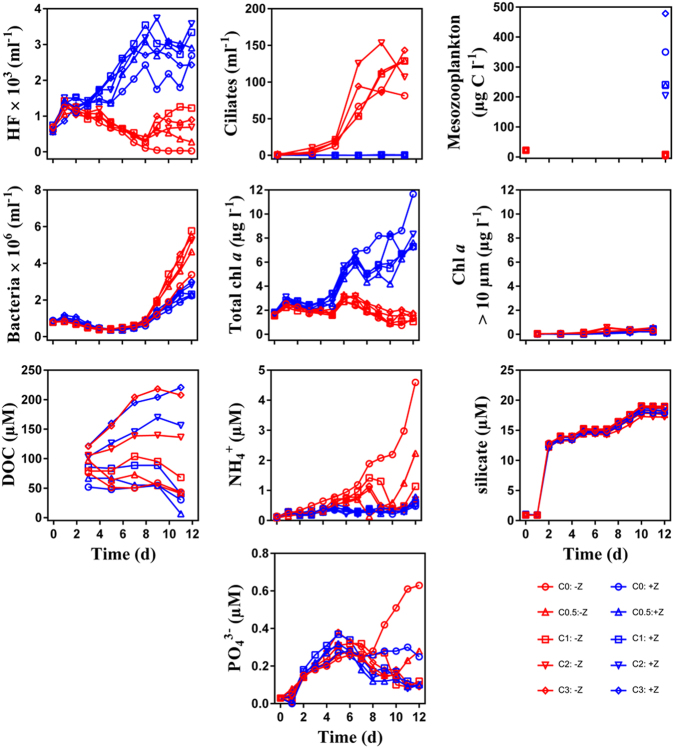
Measured standing stocks and dissolved nutrients arranged as in Fig. 2. Red and blue curves correspond to –Z and +Z treatments, respectively. The glucose gradients (0, 0.5, 1, 2 and 3 × Redfield) are represented by the symbol sequence (○, Δ, □, ∇, ◊)

Silicate concentrations increased in all tanks from 0.9 ± 0.0 µM to 18.2 ± 0.6 µM in the end of the experiment (Fig. 3). In + Z tanks ammonium (NH_4_^+^) concentrations remained at levels <0.8 µM but increased in –Z tanks until the end of the experiment with peaks of 4.6 µM, 2.2 µM, 1.1 µM, 0.6 µM and 0.6 µM in the 0C, 0.5C, 1C, 2C, 3C tanks, respectively. Concentration of PO_4_^3−^ increased tenfold in all tanks from 0.03 µM until day 5 and then decreased in all tanks with C additions to 0.10 µM. Tanks with 0C and 0.5C additions were exceptions, there PO_4_^3−^peaked at 0.63 µM in –Z: 0C, 0.28 µM in –Z: 0.5C and 0.25 µM in +Z: 0C tanks. DOC concentrations were similar in tanks with the same C additions and increased from day 3 onwards in 2C and 3C tanks from 121 µM to 214 µM and 104 µM to 146 µM, respectively. In the tanks with 1C, 0.5C and 0C additions, concentrations decreased to 82 µM, 59 µM and 30 µM, respectively.

### Bacterial activity and diversity

**Fig. 4:**
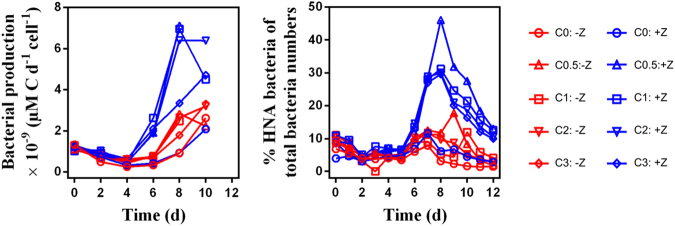
**a** Bacterial production per cell. **b** Relative abundance of the subgroup of HNA bacteria (=“Winnie-the Pooh” strategists) of total bacterial abundances. The glucose gradients (0, 0.5, 1, 2 and 3×Redfield) are represented by the symbol sequence (○, Δ, □, ∇, ◊)

Initial bacterial production was 1.2 ± 0.1 × 10^−9^ µM C d^−1^ cell^−1^ and decreased in all tanks until day 4 to rates less than half the initial value (Fig. 4a). In –Z tanks bacterial production per cell increased from day 6 onwards to a maximum of 2.8 ± 0.5 × 10^−9^ µM C d^−1^ cell^−1^, with lowest rates in tanks with 0 and 0.5 C additions. In + Z tanks with C additions bacterial production per cell increased to a peak of 6.3 ± 1.1 × 10^−9^ µM C d^−1^ cell^−1^ on day 8 and 10, and 2.1 × 10^−9^ µM C d^−1^ cell^−1^ in the tank with no C added. The peak in bacterial production per cell in + Z tanks coincides with a peak of a subgroup of HNA bacteria, detected by flow cytometry (Fig. S1). Until day 6, this group, generally considered larger and more active than LNA bacteria [41–45] made up approximately 10% of the total bacterial abundance (Fig. 4b). In + Z tanks with added C, the subgroup of HNA bacteria increased after day 6 on and peaked on day 8, when they made up 34 ± 8 % of the total bacterial abundance, in +1 C they even reached 46 %, while remaining at levels <10 % throughout the experiment in the +Z tank with no C additions. Abundances of the subgroup of HNA bacteria in –Z tanks did not exhibit pronounced changes in any of the tanks at any time in the experiment, but was lowest for the 0C tank. For comparison, the total bacterial production is presented in Figure S3: Initial average total bacterial production was 0.98 μM C L^−1^ d^−1^. From day 8 onwards total bacterial production increases, less so in the tanks with no C addition, with no marked differences between +Z and –Z or along the carbon addition gradient.

**Fig. 5:**
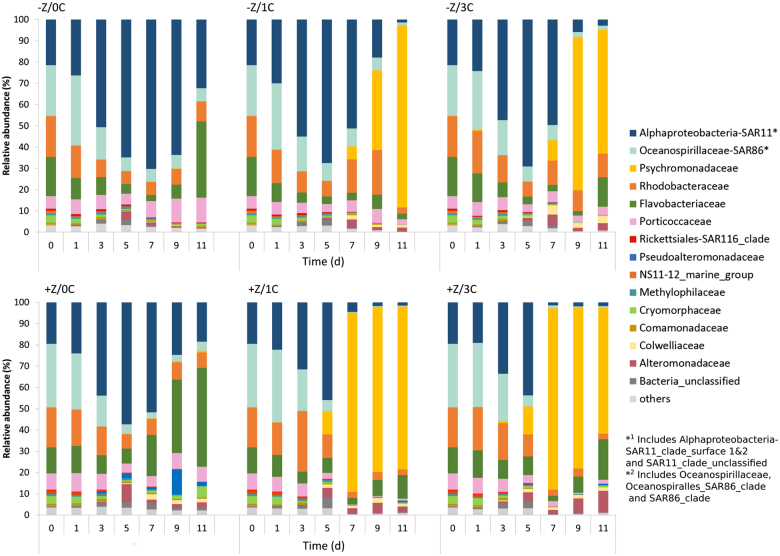
Prokaryote community composition demonstrating how the Alphaproteobacteria-SAR11-dominated community was replaced by a Psychromonadaceae dominated one in glucose-amended tanks

A total number of 3,711,093 sequence reads were received from the sequencing centre in Oslo. After quality trimming and chimera removal, the complete 16S rRNA gene dataset comprised 3,436,777 reads from 6 tanks, with an average number of 572,796 reads/ tank. The bacterial community was dominated by typical marine taxa including the Alpha- and Gammaproteobacteria and Flavobacteria. The relative abundance of these taxa varied over time and with the treatments. A succession was seen with organisms affiliated to Oceanospirillaceae-SAR86 (which includes Oceanospirillaceae, Oceanospiralles_SAR86_clade and SAR_86_clade) being most predominant at the beginning of the experiment, followed by Alphaproteobacteria-SAR11(which includes Alphaproteobacteria-SAR11_clade_surface 1&2 and SAR11_clade_unclassified) (Fig. 5). On day 5, Alphaproteobacteria-SAR11 were dominating in all mesocosms, but declined from then on. We then observed a different trend in the tanks with added glucose, where a distinct community shift was seen towards the predominance of Psychromonadaceae. In the + Z tanks Psychromonodaceae increased from 11 and 13%, on day 0 for +Z:1C and +Z:3C respectively, to >80% of the sequence reads. In –Z tanks, the shift towards Psychromonadaceae- dominance occurred later. Tanks without glucose addition, shifted towards a predominant Flavobacteriaceae (>30%) population.

## Discussion

Our experimental results were in good agreement with the dynamics of our minimum model (Fig. 2). However, in the treatments with high copepod levels and added carbon, observed bacterial density, production per cell, and ability to consume glucose were much higher than predicted by the model. We argue that this discrepancy can be traced back to internal changes in the bacterial community, not represented in the minimum model.

Initially Alphaproteobacteria-SAR 11, a group capable of utilising glucose at low concentrations [57], and Oceanospirillaceae, whose members are able to degrade complex organic compounds [58], dominated the bacteria community. In the tanks amended with glucose and copepods, Psychromonadaceae, which have a known preference to glucose as a C source [59], replaced these taxa. This switch in community composition, not observed in the 0C-tank, was a clear response to the organic carbon addition. Flavobacteria have an ability to break down complex organic matter by direct attachment and exo-enzymatic attack of algal cells and algal-derived detrital particles [60–62] and are therefore characterised as important ‘first responders’ to phytoplankton blooms. They were recently connected to phytoplankton blooms in Arctic waters [63]. The observed increased contribution of Flavobacteria to total community diversity when no organic carbon was supplied, can thus be seen as a typical response to environmental conditions where the bacteria are dependent on an autochthonous organic carbon source, in this case provided by the blooming phytoplankton.

The shift in bacterial community composition in the presence of glucose occurred for both zooplankton treatments. In the presence of copepods, however, the Psychromonadaceae-dominance occurred earlier than when copepods were absent, suggesting a stronger selection for these under high flagellate grazing. In contrast to model predictions, we observed higher prokaryote production per cell in +Z: +C than in –Z: +C, even though no pattern was detected in the total BP. The higher production combined with the lower bacterial abundance in the +Z: +C treatments imply that per-cell activity was also higher. This seems to indicate higher protein synthesis per cell in +Z treatments, but may in principle also mean a reduced isotope dilution. The leucine-based bacterial production estimates are calculated with the same dilution factor (=1) in both cases. As we have used also a fixed conversion factor of leucine to carbon, bacterial C-production in mesocosms with particularly C-rich bacteria would be underestimated.Higher copepod density thus seemed to have a bottom-up effect on the prokaryotes, although opposite to that predicted by the minimum model.

Contrary to the original model, we also found little effect of the +: −Z manipulations on measured DOC levels (Fig. 3). Combining the higher leucine-based estimates for bacterial production in +Z treatments with an assumption of constant yield would lead to an expectation of higher consumption and therefore lower DOC concentrations in the +Z treatments. As these calculations are influenced by both the leucine – carbon conversion factor, bacterial respiration, and allochthonous DOC production, it is difficult to separate the underlying mechanisms.

We also observed an increase of a subgroup of HNA bacteria, shown to be larger in size [38] and more active [64] than LNA bacteria [42]. The abundance of the HNA bacteria subgroup increased slightly with glucose addition, but the main effect was due to copepod manipulations, with high abundances of the subgroup of HNA bacteria in +Z treatments (Fig. S2). Assuming larger bacteria to be less edible by heterotrophic flagellates, this predation effect fits reports where experimentally increased predation pressure selects for inedible bacteria [65–67]. The same effect has been shown to be enhanced under phosphate-limited (glucose-replete) conditions [68]. Cells that use excess glucose to increase size will, theoretically, have a potential to increase their competitiveness for mineral nutrients by increasing their surface without simultaneously increasing their cell quota of limiting mineral nutrients. Based on these observations, we suggest that the successful Psychromonadaceae belong to what has been proposed as “Winnie-the-Pooh” strategists [69]: organisms that use a non-limiting substrate to simultaneously increase competitiveness and defence.

**Fig. 6:**
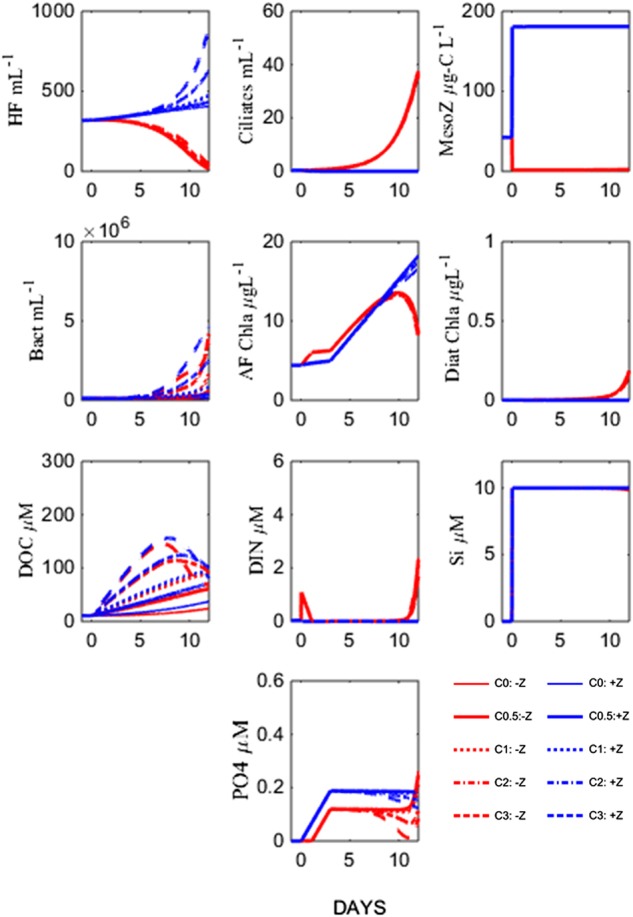
Model run with two groups of bacteria, one group with the same parameters as in the initial model and one Winnie-the-Pooh strategist group with reduced clearance rate (from heterotrophic flagellates) and increased mineral nutrient affinity when free glucose is present (see SI for details). Thereby, the difference in modelled bacterial abundance and DOC levels between the +Z and –Z levels is reduced compared to the initial model run (Fig. 2). The blue and red lines represent Z+ and Z− treatments, respectively

To illustrate the effect of such a bacterial strategy on ecosystem functioning, we added an extra group of bacteria to the minimum model (Fig. 6, Fig. S1). This group mimics the Winnie-the-Pooh strategy by simultaneously reducing clearance rate (from heterotrophic flagellates) and increasing mineral nutrient affinity when free glucose is present (see SI and Fig S1 for details). With this modification, the difference in modelled bacterial abundance and DOC levels between the +Z and –Z levels is reduced (Fig. 6); bringing model results closer to the corresponding experimental results. Interestingly, the need for more than one bacterial PFT was speculated when originally developing the minimum model for a mesocosm experiment with glucose addition in a Danish fjord [24], where the authors argued that the one-PFT bacterial representation could not simultaneously reproduce both the level and the pattern observed in bacterial production.

The Atlantic Arctic and Atlantic subarctic areas (*sensu* [70]) display among the highest mean daily POC fluxes in the world oceans [71]. The fluxes are strongly connected to the planktonic community composition in these areas [72] with commonly occurring diatom and *Phaeocystis* blooms, accounting for the highest carbon export potentials. With the ≥1 year needed for the reproductive cycle of Arctic copepods [73], the trophic cascade observed in our experiment could well reflect the response of the upper ocean when copepods rise in the water column after the winter diapause. As a response to increased predation pressure in +Z the bacterial community diversified faster and invested resources into defence against predators, through increase in cell size. With high abundance of copepods, HNF and primary producers (Fig. 3), more carbon ends up in the POC pool, therefore suggesting higher export potential through the BP.

In –Z, ciliates rapidly become the top predators and via the absence of nanoflagellates and large autotrophic flagellates the microbial loop remains active in converting carbon into more refractory forms. However the same lack of larger autotrophs and predators suggests that particulate carbon export will be reduced in this setup, rendering bacterial consumption of organic carbon a more major pathway for carbon cycling

Where ciliates are the key predators (−Z), nutrient cycling processes continue within the microbial loop but the absence of flagellates and large autotrophs means the connection to higher trophic levels is severed. Accumulation of particulate organic material will then be lower and bacterial degradation of carbon would become a more important pathway for carbon cycling in this system. As bacterial degradation of carbon has been found to produce mostly recalcitrant forms [74] we hypothesise that even though the DOC supply is high, more carbon would be sequestered into dissolved forms than moving up the food chain, where it would end up contributing more to the BP. Thus with copepods absent or arriving later in the season, we could observe the counterintuitive scenario that more organic carbon supply in the photic zone gives less particulate organic carbon accumulation within the microbial food web [75].

Published work from the Arctic, indicates substantial and often rapid changes in top predator community (e.g. [76–78]) with subsequent effects on community production. Our results suggest that changing patterns of top-down predation and carbon supply in the Arctic can have important consequences on how carbon is allocated in the food web and how modifications in life strategy influence consumption and allocation patterns.

## Electronic supplementary material

Supplemental information for Tsagaraki et al.
