# Supplementary material for: Bacterial community composition responds to changes in copepod abundance and alters ecosystem function in an Arctic mesocosm study
Source: ISME J. 2018 Jul 10;12(11):2694–705. doi: 10.1038/s41396-018-0217-7 (PMC6194086; doi:10.1038/s41396-018-0217-7)
Supplement: Supplementary file 1 — Supplemental information for Tsagaraki et al. [file 41396_2018_217_MOESM1_ESM.docx]

Supplementary information

**Bacterial community composition responds to changes in copepod abundance and alters ecosystem function in an Arctic mesocosm study**

Tsagaraki et al

**Model description**

*Minimum model:* The differential equation formulation of the minimum model used here is very close to the original formulation, developed to analyse the results of a mesocosm experiment in a Danish fjord (Thingstad et al 2007) and subsequently used by Larsen et al (2015) for two mesocosm experiments in the same Arctic location as the experiment reported here. The core set of differential equations describes the stocks and flows of a phosphorus (P)-budget. The initial conditions of the P-cycle are calculated by assuming initial values for total-P (initial *P_T_* = 220 nM-P) and a P-content in the mesozooplankton standing stock (initial M = 70 nM).

Initial values for the other P-variables are calculated as functions of *P_T_* and *M* by solving the set of algebraic equations that originate from assuming 1)steady state in the microbial part of the P-cycle and 2)Holling type I (linear) relationships between food concentration and food uptake (see Thingstad et al. 2007 for details). The model is run with hyperbolic (Holling Type II) relationships. Our philosophy is to minimize tuning of the original set of parameters, however, where changes have been made; the previous value (from Larsen et al. 2015) is given in parenthesis (Table S1).

Conversion from the P-units of the basic model to measured units (abundances, chlorophyll, carbon, nitrogen) are done with fixed conversion factors (Table S1). The associated C-cycle is highly simplified, but involves two important assumptions, both based on fitting the model to the results of the Danish fjord experiment (Thingstad et al. 2007): 1) Primary production in flagellates is proportional to P-uptake in autotrophic flagellates, while diatom primary production is proportional to diatom biomass. 2) The presumably complex set of mechanisms responsible for autochthonous production of labile DOC is parameterized as a production rate proportional to the square of total-P. The model has no representation of recalcitrant DOC.

The Danish fjord experiment used for original model fitting had water temperatures ranging between 15.3 and 17.9 ^o^C (Havskum et al. 2003). In the experiment reported here, water temperatures varied between 5 and 12 ^0^C, average 7.2 ^o^C. Microbial population dynamics in previous experiments at ca 7 ^o^C in the same Arctic location was only slightly retarded relative to the Danish experiment, suggesting a moderate temperature correction with Q_10_≈ 1.4 (Larsen et al. 2015). Original rate parameters are therefore divided by 1.4.

*Addition of a nitrogen budget to the minimum model (Fig 1):* To reproduce ammonium values in this N-deficient system, a simple representation of the N-cycle is coupled to the P-cycle. All 6 populations have a fixed N:P stoichiometry and free ammonium is calculated as the difference between total N and N bound in biomass. Ammonium dependence of osmotrophs growth rates are included as multiplicative hyperbolic terms with affinity for ammonium equal to affinity for phosphate divided by the group’s N:P-ratio.

*Addition to minimum model with “Winnie-the-Pooh” bacteria included (Fig.7):* To illustrate the theoretical effect of a community change, we added an extra bacterial plankton functional type, having the same properties as the original bacteria, but changing its nutrient affinity and clearance rate by heterotrophic flagellates at elevated DOC concentrations.

In the original minimum model, the differential equation for bacteria (*B*) is

$$\frac{dB}{dt}=\left( \mu_{B}-\delta_{B} \right)B$$

Where *µ_B_* and *δ_B_* are the specific (per unit *B*) growth and loss rates, respectively, for bacteria.

Bacterial growth rate is the product of the three hyperbolic terms for phosphate (P), ammonium (N) and labile organic-C (L) :

$$\mu_{B}\left( P,N,L \right)={\mu_{B}^{max}}^{-2}\cdot\frac{\alpha_{BP}\cdot P}{1+\frac{\alpha_{BP}\cdot P}{\mu_{B}^{max}}}\cdot\frac{\alpha_{BN}\cdot N}{1+\frac{\alpha_{BN}\cdot N}{\mu_{B}^{max}}}\cdot\frac{\alpha_{BL}\cdot L}{1+\frac{\alpha_{BL}\cdot L}{\mu_{B}^{max}}}$$

Where α_BN_ = α_BP_^/^ν, where ν=8 is the molar N:P ratio of bacteria.

Loss rate δ_B_ is the predation loss to heterotrophic flagellates (H), expressed as:

$$\delta_{B}=\frac{\alpha_{HB}H}{1+\frac{(B+s\cdot WB)}{I_{H}^{max}}}$$

Where WB are the Winnie-the-Pooh bacteria, σ_WB_ the selectivity factor of H for WB (see below), and $I_{H}^{max}$ is maximum specific ingestion rate for H*.*

When WB are present, two dimensionless factors are calculated as functions of L. These are used to modify WB’s nutrient affinity and resistance to grazing, relative to B.

$$f=0.5+4\cdot(1-e^{-\frac{L}{{10}^{4}}})$$

$$s=\frac{1}{1+3\cdot(1-e^{\frac{-L}{{10}^{4})}}}$$

Growth and loss rate for WB are then calculated as for B, but with mineral nutrient affinities multiplied with *f* and clearance rate multiplied with *s.*

The expressions for *f* and *s* are chosen to give monotonically increasing *f*  and *s*, respectively (Fig S1). Also,  *f* < 1 and *s* = 1 for *L*=0 to make WB competitively inferior and without grazing resistance when there is no excess of labile DOC.

The numerical model is coded in Matlab^®^, available on request to [frede.thingstad@uib.no](mailto:frede.thingstad@uib.no).

Molecular analysis of bacterial community

After sample processing the filters were capped and frozen in liquid nitrogen, shipped to Bergen on dry ice and stored at -80°C until further processing. DNA was extracted from the filters using the AllPrep DNA/RNA Mini Kit (Qiagen, Ca, USA) and the manufacturer’s protocol. DNA quantity was measured using the Qubit 3.0 Fluorometer (Thermo Fisher Scientific inc., Ma, USA) and quality was assessed by agarose gel electrophoresis.

The V4 region of the 16S rRNA gene was amplified using a two-step protocol. In the first step triplicate samples were amplified using primer combination of 519F (5’-CAGCMGCCGCGGTAA-3`) (Øvreås et al 1997) and a modified 806RB primer (5`- GGACTACNVGGGTWTCTAAT-3`) as described by Apprill et al 2015. The reaction was carried out in 20 µl volume containing 10µl HotStarTaq MasterMix (Qiagen), 0,5 µM of each primer and 1-10 ng of DNA template. The amplification was carried out with an initial denaturation for 15 min at 95°C, followed by 25 cycles of denaturation at 95°C for 20 seconds, annealing at 55°C for 30 sec and extension at 72°C for 30 sec, with a final extension at 72°C for 7 min. The triplicate amplicons were pooled into a single volume and purified using the Zymo DNA clean and concentrator kit (Zymo Research Corporation CA, USA). Pooled amplicons were subjected for a second PCR using MID tagged primers 519F and 806RB using the same conditions as before except that the annealing temperature was set to 62°C and a total of 15 cycles were run. The final PCR products were purified using Agencourt AMPure XP magnetic beads (Beckman Coulter inc. CA, USA) and checked for quantity and quality as described above. Amplicons were then pooled in equal amounts prior to library construction. The samples were sent to the Norwegian Sequencing Centre (Oslo, Norway) for high throughput sequencing using the MiSeq Platform with Reagent Kit v2 (Illumina, CA, USA).

**Mesocosm filling and copepod colection**

One day before the experiment started, the tanks were filled at a distance from shore (78.56N, 11.58E) and from below the pycnocline (6-7m depth) to avoid silt and clay containing riverine inflow. A submersible centrifugal pump was used, with no metal parts in contact with the water-flow. Water was pumped sequentially into the tanks at timed intervals, to minimise differences due to seawater patchiness and during filling was screened through a 200µm net to remove larger zooplankton. The tanks were then towed to port and moored on the pier as described in Larsen et al (2015).

Copepods for the addition to the tanks at start were collected by horizontal tows at ~30 meters depth using a plankton net with mesh size 500 µm (diameter 1.5 m, length 10 m) with a non-filtering cod end. The collected copepods were kept in 100 L buckets overnight and non-viable copepods were allowed to sink to the bottom and removed by siphoning.

Table S1. Symbols, parameter values and conversion factors used in the minimum model.

| **Symbol** | **Description** | **Initial value** | **unit** |
| --- | --- | --- | --- |
| *State variables* | | |  |
| *B* | Biomass bacteria | calculated | nmol-P L^-1^ |
| *A* | Biomass autotrophic flagellates | calculated | nmol-P L^-1^ |
| *D* | Biomass diatoms | Calculated, if steady state value 0 then set to 0.1 | nmol-P L^-1^ |
| *H* | Biomass heterotrophic flagellates | Calculated | nmol-P L^-1^ |
| *C* | Biomass ciliates | Calculated | nmol-P L^-1^ |
| *M* | Biomass mesozooplankton | 70 | nmol-P L^-1^ |
| *P* | Free bioavailable phosphate | Calculated | nmol-P L^-1^ |
| *L* | Bioavailable dissolved organic carbon (BDOC) |  | nmol-C L^-1^ |
| *S* | Free silicate |  | nmol-Si L^-1^ |
|  |  |  |  |
| *Affinities and clearance rates* | | **Parameter value** |  |
| α*_BP_* | Bacterial affinity for *P* | 0.08 | L nmol-P^-1^ h^-1^ |
| α*_BL_* | Bacterial affinity for *L* | 1x10^-6^ (5.3x10^-6^ _)_ | L nmol-P^-1^ h^-1^ |
| *α_AP_* | Affinity of autotrophic flagellates for *P* | 0.04 | L nmol-P^-1^ h^-1^ |
| *α_DP_* | Diatom affinity for *P* | 0.03 | L nmol-P^-1^ h^-1^ |
| *α_DS_* | Diatom affinity for *S* | 0.0012 | L nmol-P^-1^ h^-1^ |
| *α_H_* | Heterotrophic flagellate clearance rate for bacteria | 0.0015 | L nmol-P^-1^ h^-1^ |
| *α_C_* | Ciliate clearance rate for auto- and heterotrophic flagellates | 0.0005 | L nmol-P^-1^ h^-1^ |
| *α_M_* | Mesozooplankton clearance rate for diatoms | 0.00015 | L nmol-P^-1^ h^-1^ |
| $\sigma$ | Mesozooplankton selectivity factor for ciliates relative to diatoms | 2 | Dimensionless |
|  | | |  |
| *Maximum growth rates* | | |  |
| $\mu_{B}^{m}$ | Maximum growth rate *B* | 0.25 | h^-1^ |
| $\mu_{A}^{m}$ | Maximum growth rate *A* | 0.054 | h^-1^ |
| $\mu_{D}^{m}$ | Maximum growth rate *D* | 0.063 | h^-1^ |
| $\mu_{H}^{m}$ | Maximum growth rate *H* | 0.1 | h^-1^ |
| $\mu_{C}^{m}$ | Maximum growth rate *C* | 0.05 | h^-1^ |
| $\mu_{M}^{m}$ | Maximum growth rate *M* | 0.00625 | h^-1^ |
|  |  |  |  |
| *Yields* | | |  |
| *Y*_BC_ | Bacterial yield on *L* | 0.004 | nmol-P nmol-C^-1^ |
| *Y_BCW_* | Winnie-the-Pooh bacteria, yield on L | 0.003 | nmol-P nmol-C^-1^ |
| *Y*_H_ | Heterotrophic flagellate yield on bacteria | 0.3 | nmol-P nmol-P^-1^ |
| *Y*_C_ | Ciliate yield on auto- and heterotrophic flagellates | 0.2 | nmol-P nmol-P^-1^ |
| *Y*_M_ | Mesozooplankton yield on ciliates and diatoms | 0.15 | nmol-P nmol-P^-1^ |
|  |  |  |  |
| *Forcing parameters for microbial part of the system* | | |  |
| *ψ* | Supply rate *L* |  | nmol-C L^-1^ h^-1^ |
| *P_t_* | Total-P in microbial part of the food web | 220 (initial) | nmol-P L^-1^ |
| *S_t_* | Total-Si | Initial: 0 | nmol-Si L^-1^ |
|  |  |  |  |
| *Stoichiometric ratios and conversion factors* | | | |
| *ρ_B_* | Molar carbon:phosphorous ratio in bacteria | 50 | nmol-C nmol-P^-1^ |
|  | Molar carbon:phosphorous ratio in autotrophic flagellates | 106 | nmol-C nmol-P^-1^ |
| *ρ_DS_* | Molar phosphorous:silicate ratio of diatoms | 0.04 | nmol-C nmol-P^-1^ |
| *ρ_Chl_* | P to Chl ratio | 47.2 | nmol-P:µgChl |
|  | Molar N:P bacteria and diatoms | 8 (new) |  |
|  | Molar N:P flagellates, ciliates and mesozooplankton | 16 (new) |  |
| *ρ_BP_* | P:cell bacteria | 3.33x10^-8^ | nmol-P |
| *ρ_HF_* | P:cell heterotrophic flagellates | 1x10^-5^ (4x10^-4^) | nmol-P |
| *ρ_C_* | P:cell ciliates | 1x10^-2^ | nmol-P |
| *Others* |  |  |  |
| *γ* | Fraction of consumed diatom Si that is remineralized to free Si | 0.25 | Dimensionless |
| *ε_A_* | Autotrophic flagellate ability to turn off excess C-fixation | 1 | Dimensionless |
| *ε_D_* | Diatom ability to turn off excess C-fixation | 0 | Dimensionless |
| *k* | Proportionality constant between production of *L* and P^2^_T_ | 1.1 × 10^-4^ | nmol-C L^-1^ h^-1^ |
| *δ_M_* | Loss rate of mesozooplankton | 0 | (nmol-P L^-1^)^-2^ d^-1^ |

Figure S1 DOC-dependence of multiplication factors modifying. (f) the mineral nutrient affinity, and (s) the clearance rate of heterotrophic flagellates, for WtP-bacteria, relative to that of standard bacteria.


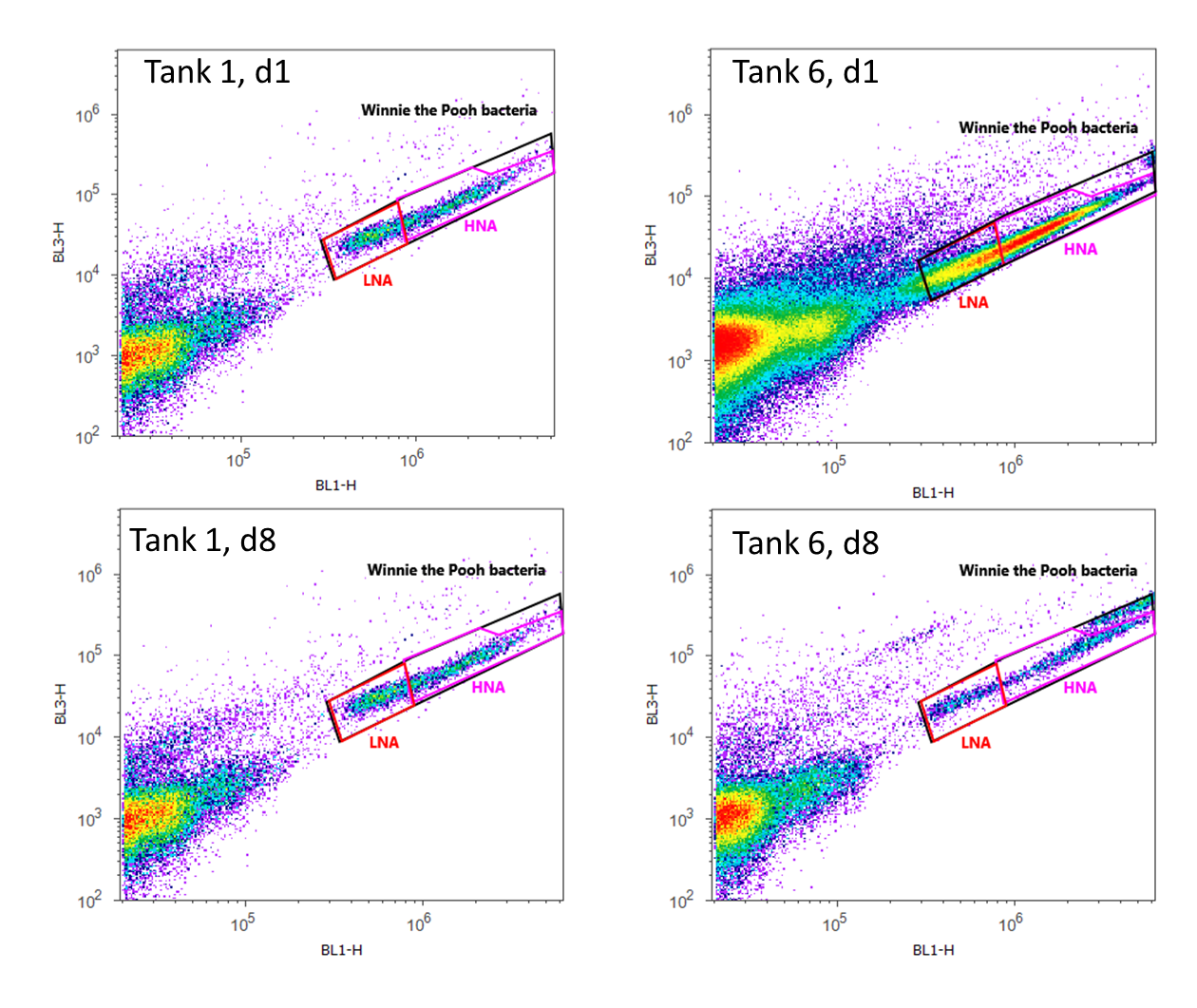


Fig. S2 Flow cytometry plots with three different bacterial populations, discriminated based on red and green fluorescence after staining with SYBR Green I of tank 1 (-Z:0C) and tank 6 (+Z:0.5C) in the beginning and on day 8 of the experiment. The stain reflects the amount of DNA and hence genome sizes: Low nucleic acid bacteria (LNA), high nucleic acid bacteria (HNA) and WtP bacteria, as a subgroup of HNA bacteria. WtP only appear as a distinct group in tank 4, day 8.


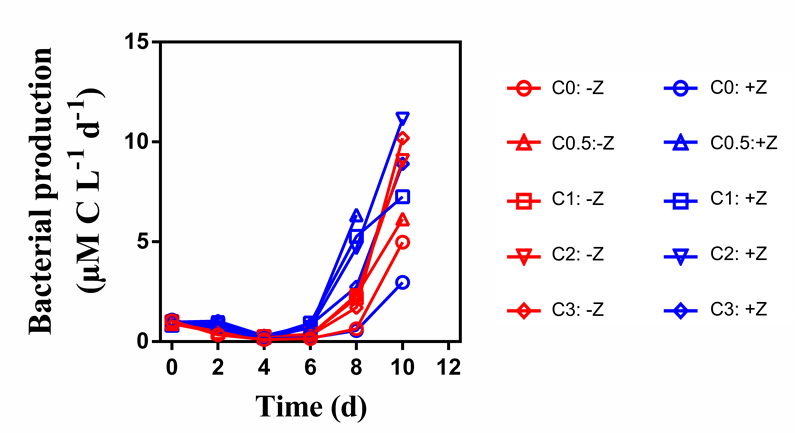


Fig. S3 Total bacterial production in during the course of the experiment. Red lines correspond to –Z tanks while blue lines to +Z tanks. C refers to glucose addition level(0.3) and –Z, +Z to the low and high grazing, respectively

References

Apprill A, McNally S, Parsons R, Weber L (2015). Minor revision to V4 region SSU rRNA 806R gene primer greatly increases detection of SAR11 bacterioplankton. Aquat Microb Ecol **75**: 129-137.

Havskum, H., et al. (2003). "Silicate and labile DOC interfere in structuring the microbial food web via algal-bacterial competition for mineral nutrients: Results of a mesocosm experiment." Limnology and Oceanography **48**(1): 129-140.

Larsen A, Egge JK, Nejstgaard JC, Di Capua I, Thyrhaug R, Bratbak G *et al* (2015). Contrasting response to nutrient manipulation in Arctic mesocosms are reproduced by a minimum microbial food web model. *Limnology and Oceanography* **60:** 360-374.

Øvreås L, Forney L, Daae FL, Torsvik V (1997). Distribution of bacterioplankton in meromictic Lake Saelenvannet, as determined by denaturing gradient gel electrophoresis of PCR-amplified gene fragments coding for 16S rRNA. Appl Environ Microbiol 63: 3367-3373.

Thingstad TF, Havskum H, Zweifel UL, Berdalet E, Sala MM, Peters F *et al* (2007). Ability of a "minimum" microbial food web model to reproduce response patterns observed in mesocosms manipulated with N and P, glucose, and Si. *Journal of Marine Systems* **64:** 15-34.
